# Epidemiology of cervical human papillomavirus (HPV) infection and squamous intraepithelial lesions (SIL) among a cohort of HIV-infected and uninfected Ghanaian women

**DOI:** 10.1186/s12885-017-3682-x

**Published:** 2017-10-16

**Authors:** Dorcas Obiri-Yeboah, Patrick K. Akakpo, Mohamed Mutocheluh, Emmanuel Adjei-Danso, Gloria Allornuvor, Daniel Amoako-Sakyi, Yaw Adu-Sarkodie, Philippe Mayaud

**Affiliations:** 1 Department of Microbiology and Immunology, School of Medical Sciences, University of Cape Coast, Cape Coast, Ghana; 2 Department of Pathology, School of Medical Sciences, University of Cape Coast, Cape Coast, Ghana; 3 Department of Clinical Microbiology, School of Medical Sciences, Kwame Nkrumah University of Science and Technology, Kumasi, Ghana; 4 Mineworkers Union of Trade Union Congress, Tarkwa, Ghana; 5 Department of Obstetrics and Gynaecology, Cape Coast Teaching Hospital, Cape Coast, Ghana; 6 Department of Clinical Research, Faculty of Infectious and Tropical Diseases, London School of Hygiene and Tropical Medicine, London, UK

**Keywords:** Human papillomavirus (HPV), Genotyping, Squamous intraepithelial lesions (SIL), Cervical cancer, Human immunodeficiency virus (HIV), Ghana

## Abstract

**Background:**

There is limited data in Ghana on the epidemiology of HPV and cervical neoplasia and their associations with HIV. This study aimed to compare among HIV-1 seropositive and HIV-seronegative Ghanaian women: (1) the prevalence, genotype distribution and risk factors associated with cervical HPV infection; and (2) the prevalence and risk factors associated with abnormal cervical cytology.

**Methods:**

A comparative frequency-matched study was conducted in a systematic sample of women aged ≥18 years attending HIV and general outpatient clinics in Cape Coast Teaching Hospital, Ghana. Participants were interviewed and cervical samples collected for HPV genotyping (Seegene Anyplex-II HPV28) and cytological testing.

**Results:**

Overall, 333 women were recruited, 163 HIV-1 seropositive and 170 HIV-seronegative women of mean age 43.8 years (SD ±9.4)) and 44.3 years (SD ±12.8), respectively. The prevalence of 14 high-risk (hr) HPV genotypes was higher among HIV-1 seropositive women (65.6% vs. 30.2%, *P* < 0.0001), as was proportion with multiple hr.-HPV infections (60.6% vs. 21.3%, *P* < 0.0001). HPV35 was the most prevalent hr.-HPV genotype in both groups (11.9% and 5.3%). The main factors associated with hr.-HPV infection were age for HIV-positive women and circumcision status of main sexual partner for both HIV-negative and positive women.

Abnormal cervical cytology prevalence was higher among HIV-1 seropositive women (any SIL: 14.1% vs. 1.2%, *P* < 0.0001; low-grade SIL [LSIL]: 4.9% vs. 0.6%, *P* = 0.02; high-grade SIL: 1.8% vs. 0%, *P* = 0.07). Among HIV-1 seropositive women, number of pregnancies and CD4+ cell count were associated with LSIL+ cytology. There was strong association between LSIL+ abnormalities and HPV35 (aOR = 4.7, 95%CI: 1.3–17.7, *P =* 0.02).

**Conclusions:**

HIV-1 infected women bear significant burden of HPV infection and related disease. Prevention and screening programmes should be specifically deployed for this population in Ghana.

**Electronic supplementary material:**

The online version of this article (10.1186/s12885-017-3682-x) contains supplementary material, which is available to authorized users.

## Background

Persistent infection with genital human papillomavirus (HPV) is causally linked with many genital cancers, including cervical cancer [1, 2]. Genital HPV genotypes are further categorized broadly into low-risk (lr) and high-risk (hr) types based on their oncogenic potential. Persistence of hr.-HPV in the transformation zone of about 10% of infected persons may lead over time to squamous intraepithelial lesions (SIL) or cervical intraepithelial neoplasia (CIN) and invasive cervical carcinoma (ICC).

Co-infection with HPV has implications for HIV-infected women and their health care providers. HIV increases the risk of HPV persistence and development of associated cervical lesions [3]. In particular, low CD4+ T-lymphocyte counts may increase the risk of recurrence [4, 5], whilst higher CD4+ cell counts may promote HPV clearance, highlighting the role of immunity in the development of cervical disease [6]. The role of antiretroviral therapy (ART) is more complex. By decreasing HIV plasma viral load and restoring immunity, ART is expected to have benefits in reducing HPV-associated clinical conditions among HIV-infected women [7–9], especially among ART users with high adherence [10]. However, several studies have not reported such benefits of ART directly [11–13]. Improved survival among women living with HIV taking ART increases potential exposure time and may lead to higher cancer rates, underscoring the need for specific screening and management programmes in this high-risk population.

Other known risk factors of HPV acquisition, persistence and development of cervical lesions include age, age at first sexual activity, life time number of sexual partners [14–16], smoking [17], hormonal contraceptive use [18, 19], co-infection with other STIs [20, 21] and lack of circumcision of the male partner [22].

Forman et al. [23] have estimated the global adjusted HPV prevalence to be 11.7%, with West Africa having the fourth highest with a prevalence of 19.6%. Limited data on HPV and SIL/CIN epidemiology are available in Ghana from women attending gynaecological clinics. Brandful et al. [24] reported an HPV prevalence of 64.5% among HIV-seronegative pregnant women in Accra. Attoh et al. [25] in their study among 50 women diagnosed with cervical cancer in Ghana found cervical HPV DNA prevalence of 98%, with HPV18 being the most predominant type (84%) followed by HPV16 (24%). Cervical cancer screening in the country is not systematically organized with some pilot implementation of the WHO-recommended visual inspection with acetic acid (VIA) [26], whilst cytological screening using the Papanicolaou (pap) method is becoming more available, albeit with associated significant costs and logistical challenges.

Since 2005, the use of HPV vaccines, first the bivalent and quadrivalent vaccines targeting HPV16 and 18 linked to 70% of invasive cancers (and low risk HPV 6 and 11), and recently the nonavalent vaccine targeting the same types plus five other hr. types (HPV31, 33, 45, 52 and 58), globally linked to 90% of cancers [27], has made primary prevention possible. These vaccines have been shown to be effective in preventing HPV infection and the development of genotype-specific HPV-associated clinical conditions [28–30]. In Ghana, a pilot HPV vaccination programme targeting school-going girls aged 9–13 was carried out in 2013 in selected regions using the quadrivalent vaccine. More detailed information on the prevalence and distribution of HPV genotypes associated with lesions in various patient groups in Ghana would help inform HPV vaccination and screening plans.

The objectives of this study were to compare between HIV-1 sero-positive and HIV sero-negative women in the Cape Coast Metropolis of Ghana: (1) the prevalence, genotype distribution and risk factors associated with HPV infection, and (2) the prevalence and risk factors associated with abnormal cervical cytology (ASCUS+).

## Methods

### Study design and subjects

This study was a comparative frequency-matched study conducted among women ≥18 years attending the HIV or medical outpatient clinic of the Cape Coast Teaching Hospital (CCTH) in Ghana. A systematic sampling of every 5th woman was used starting with a random sampling of the first woman. Women who were ineligible (previous total hysterectomy, pregnant or menstruating on that day), had the opportunity passed on to the next until a total of 10 women per day were selected. After recruitment, the study protocol was explained to each woman and written informed consent was then obtained.

Enrolled women then had their HIV status confirmed and then they answered a questionnaire in either English or the local language (Fante). This questionnaire gathered socio demographic characteristics of the women and then for HIV positive women, the HIV specific characteristics including use of ART, nadir CD4+ T-lymphocytes count, and WHO clinical staging were also obtained (Additional file 1).

### Sample collection

Blood was drawn to perform HIV serology using the First Response Kit (Premier Medical Corporation Limited, India) to detect HIV-1 and HIV-2 antibodies, confirmed with the OraQuick kit (OraSure Technologies, USA), as per national guidelines. Counseling based on the results was done for all women and women found HIV-1 seropositive but not already in care were referred to the ART treatment center at CCTH. Participants underwent gynaecological examination with speculum, during which cervical swabs were collected from the ecto/endocervix targeting the squamo-columnar junction using a DNA PAP™ Cervical Sampler™ and transported in the Swab Specimen Collection Kit (Qiagen, Gaithersburg, MD). Cervical smears were taken for cytology with a cervical brush and alcohol-fixed at the clinic. All women found to have LSIL+ cytology were referred to the gynaecologist for further evaluation and management.

### HPV DNA detection

HPV genotyping was performed using the recently developed Anyplex™ II HPV28 assay (Seegene, Seoul, Korea). The assay detects 28 HPV genotypes including 19 h-HPV types, of which 13 are considered carcinogenic (HPV16, 18, 31, 33, 35, 39, 45, 51, 52, 56, 58, 59, 68) and 6 possible carcinogenic (HPV26, 53, 66, 69, 73, 82), and 9 low-risk HPV types (HPV6, 11, 40, 42, 43, 44, 54, 61, 70), according to the Interagency for Research on Cancer (IARC) [31]. This assay has been compared with the Digene HC2 HPV DNA assay (Qiagen) and found to be analytically more sensitive in detecting the 13 h-HPV types identifiable by both assays, in addition to having higher concordance with comprehensive genotyping based on sequencing analysis [32].

The isolation of nucleic acid was done by QIAamp DNA Mini kit (Qiagen, USA) following manufacturer’s instructions. Extracted DNA samples were aliquoted and stored at -20 °C. The next step was the multiplex real time PCR using the Anyplex™ II HPV28 (Seegene, Korea) following manufacturer’s protocol with the CFX96™ Real-time PCR System (Bio-Rad, USA). In brief, this involved preparation of a master mix which contained RNAase free water (5 μl per sample), 4X Anyplex solution (5 μl per sample) and 4X HPV28 TOM A or TOM B solutions (5 μl per sample for each type). TOM A contained the primers for hr.-HPV genotypes, while TOM B contained primers for non hr.-HPV types. Both reactions were set up to run concurrently. To 15 μl of reagents was added 5 μl of the prepared DNA sample.

A set of 3 external positive controls and a negative control were included in each set to run with the samples. To be valid for any sample, both the internal and external controls must have expected result. When the external controls failed the whole set was invalid and no results were read. When the internal control for a sample failed, whilst all external controls were correct, that particular sample result was considered invalid and it tested again.

### Cervical cytology

Following a standardized protocol for Papanicolaou (pap) staining, cervical smears were prepared in the laboratory and read by a consultant cytopathologist at CCTH using the Bethesda 2001 guidelines and the LAST guidelines [33, 34].

### Sample size and statistical analysis

Sample size was calculated to allow comparisons of HIV-1 seropositive and HIV-seronegative women. Assumptions on HPV and cytological abnormality prevalence in each group were based on findings from different studies around the Africa region and sample size was powered to detect differences in the lowest frequency outcome (cytological abnormalities). Data analyses were performed using Stata version 13 software (STATA Corp, Texas USA).

A descriptive analysis of socio-demographic, behaviour and other relevant characteristics of the study population was done according to HIV serostatus. *P*-values were used to compare the parameters between the groups based on student’s *t-test* for continuous variables or chi- square test for categorical variables. Bivariate analysis was done separately for the two study outcomes (HPV and cytological abnormalities) stratified by HIV serostatus.

Based on outcome frequency, associations are presented as prevalence or risk ratios (PR/RR) with 95% confidence intervals (CI) for the HPV outcomes, and odds ratios (OR) and 95%CI for the cytological abnormality outcomes. Variables with *P*-values ≤0.20 and a priori factors like age were put in the model for multivariate analysis.

## Results

### Study participants

A total of 333 (163 HIV-1 seropositive and 170 HIV-seronegative) women were recruited between July and December 2014. Participant characteristics are shown in Table 1. The mean age of participants was 43.8 (SD ±9.4) years for HIV-1-seropositive women and 44.3 years (SD ±12.8) for HIV-seronegative women. Compared to HIV-seronegative women, HIV-1 seropositive women were less educated, more frequently in unskilled occupations, without a current partner but with a larger number of lifetime partners. Smoking was rare in this population (about 2% overall) and hormonal contraceptive history infrequent (39.3% and 42.4%, in HIV-1 seropositive and HIV-seronegative women, respectively). Most male sexual partners (93%) were circumcised, as expected in Ghana (Table 1).

**Table 1:** Characteristics and laboratory findings of enrolled participants in Cape Coast, Ghana

VARIABLES	HIV-1 seropositive (*N* = 163)	HIV seronegative (*N* = 170)	*P*-value
	n (%), or mean/median (SD or IQR)	n (%), or mean/median (SD or IQR)	
Age, years			0.03
Mean (SD)	43.8 (9.4)	44.3 (12.8)	
≤ 29	11 (6.8)	21 (12.4)	
30–59	142 (87.1)	129 (75.9)	
> 60	10 (6.1)	20 (11.8)	
Marital status			<0.0001
Currently with a regular sexual partner	70 (42.9)	126 (74.1)	
Currently without a regular sexual partner	93 (57.1)	44 (25.9)	
Education			<0.0001
No formal education	40 (24.5)	28 (16.5)	
Up to secondary school level	117 (71.8)	75 (44.1)	
Tertiary level	6 (3.7)	67 (39.4)	
Employment			<0.0001
Unemployed	14 (8.6)	14 (8.2)	
Unskilled occupation	137 (84.1)	89 (52.4)	
Skilled occupation	12 (7.4)	67 (39.4)	
Age at first sex, years Categorical			0.16
Median (IQR)	18 (17–20)	19 (18–21)	
≤ 15	7 (4.3)	6 (3.5)	
16–20	127 (77.9)	119 (70.0)	
≥ 21	29 (17.8)	45 (26.5)	
Lifetime no. sexual partners			0.004
Median	3 (2–4)	3 (2–3)	
1	11 (6.8)	31 (18.2)	
2–5	139 (85.3)	131 (77.1)	
> 5	13 (8.0)	8 (4.7)	
Circumcision main/current partner			0.90
Circumcised	153 (93.9)	159 (93.5)	
Not circumcised	10 (6.1)	11 (6.5)	
Smoking			0.09
Ever/Current	5 (3.1)	1 (0.6)	
Never smoked	158 (96.9)	169 (99.4)	
Hormonal contraception			0.57
Ever/Current	64 (39.3)	72 (42.4)	
Never used	99 (60.7)	98 (57.6)	
Cytology			<0.0001
Normal	140 (85.9)	168 (98.8)	<0.0001
ASCUS	12 (7.4)	1 (0.6)	0.001
LSIL	8 (4.9)	1 (0.6)	0.02
HSIL/ASC-H	3 (1.8)	0 (0)	0.07
HPV DNA	120/160 (75.0)	72/169 (42.6)	<0.0001

*SD* Standard Deviation, *IQR* Interquartile Range, *ASCUS* Atypical Squamous Cells of Undetermined Significance, *LSIL* Low Grade Squamous Intraepithelial Lesions, *HSIL* High Grade Squamous Intraepithelial Lesions, *ASC-H* atypical squamous cells cannot rule out HSIL

The majority (57.1%) of HIV-1 seropositive participants had been diagnosed with HIV less than 5 years ago with a median duration since HIV diagnosis of 4.3 years (interquartile range [IQR], 1.9–7.1). Most (79.1%) were taking ART, 62% for longer than 2 years. The median nadir CD4+ count of women on ART and ART-naïve was 202 cells/mm^3^ (IQR, 96–289) and 460 cells/mm^3^ (IQR, 378–560), respectively (Table 2).

**Table 2:** Clinical characteristics of 163 HIV-1 seropositive women at enrolment

VARIABLES	n (%) or median (IQR)
Duration of HIV diagnosis, years (*N* = 163)	
Median	4.3 (1.9–7.0)
< 5	93 (57.1)
≥ 5	70 (42.9)
ART status (*N* = 163)	
Not on ART	34 (20.9)
≤ 2 years	28 (17.1)
> 2 years on ART	101 (62.0)
WHO clinical stage (*N* = 159)	
Stage I & II	80 (50.3)
Stage III & IV	79 (49.7)
Nadir CD4+ count, cells/mm^3^ (*N* = 155)	
Median	246 (125–398)
< 200	63 (40.7)
200–349	49 (31.6)
350–500	22 (14.2)
> 500	21 (13.6)

*ART* antiretroviral therapy, *IQR* interquartile range

### Prevalence of HPV, genotype distribution and risk factors for hr.-HPV

A total of 329/331 obtained samples were successfully genotyped using the Seegene Anyplex II HPV28 protocol, with two samples (0.6%) giving invalid results. The overall HPV DNA prevalence was 75% (120/160) among HIV-1 seropositive women and 42.6% (72/169) among HIV-seronegative women (*p* < 0.0001). Compared to HIV-seronegative women, HIV-1 seropositive women had a higher prevalence of hr.-HPV genotypes (65.6% vs. 30.2%, *P* < 0.0001), multiple HPV infections (60.6% vs. 21.3%, *P* < 0.0001), HPV16 and/or 18 infection (21.3% vs. 2.4%, *P* < 0.0001), or infection with hr.-HPV types included in the nonavalent vaccine (i.e. HPV16/18/31/33/45/52/58/6/11) (55.6% vs. 17.2%, *P* < 0.0001) (Table 3). The most prevalent hr.-HPV genotypes among HIV-1 seropositive women in this study were HPV35 (11.9%), 52 (11.9%), 58 (11.3%), 16 (10.6%) and 18 (10.6%), whereas among HIV-negative women the most prevalent types were HPV35 (5.3%), 58 (4.1%), 33 (4.1%), 56 (3.6%), and 52 (2.4%) (Table 3).

**Table 3:** HPV prevalence and genotype distribution among HIV-1 seropositive and HIV-seronegative women in Cape Coast, Ghana

HPV genotypes	HIV-1 positive (*N* = 160)	HIV negative (*N* = 169)	*P-*value
	n (%)	n (%)	
HPV 16	17 (10.6)	1 (0.6)	<0.0001
HPV 18	17 (10.6)	3 (1.8)	0.001
HPV 31	13 (8.1)	1 (0.6)	0.001
HPV 33	8 (5.0)	7 (4.1)	0.71
HPV 35	19 (11.9)	9 (5.3)	0.03
HPV 39	7 (4.4)	3 (1.8)	0.17
HPV 45	10 (6.3)	2 (1.2)	0.01
HPV 51	2 (1.3)	0 (0.0)	0.14
HPV 52	19 (11.9)	4 (2.4)	0.001
HPV 56	13 (8.1)	6 (3.6)	0.07
HPV 58	18 (11.3)	7 (4.1)	0.01
HPV 59	8 (5.0)	2 (1.2)	0.04
HPV 68	15 (9.4)	3 (1.8)	0.002
Any HPV	120 (75.0)	72 (42.6)	<0.0001
Any hr.-HPV	105 (65.6)	51 (30.2)	<0.0001
HPV 16/18 (bivalent vaccine types)	34 (21.3)	4 (2.4)	<0.0001
HPV 16/18/6/11 (quadrivalent vaccine types)	53 (33.1)	7 (4.1)	<0.0001
HPV 16/18/31/33/45/52/58/6/11 (nonavalent vaccine types)	89 (55.6)	29 (17.2)	<0.0001
Low risk types only	15 (9.4)	21 (12.4)	0.38
HPV 6	12 (7.5)	1 (0.6)	0.001
HPV 11	10 (6.3)	2 (1.2)	0.01
HPV 6 and/or 11	21 (13.1)	3 (1.8)	<0.0001
Multiple types			
2–5	64 (52.5)	36 (21.3)	<0.0001
> 5	13 (8.1)	0 (0.0)	<0.0001

Overall, hr.-HPV prevalence was strongly associated with HIV-1 serostatus (PR = 2.2, 95%CI: 1.7–2.8). Table 4 show details of the bivariate analyses for hr.-HPV infection. Among HIV-1 seropositive women the main risk factor for hr.-HPV was age and male partner’s lack of circumcision; among HIV-seronegative women, the main risk factor was the male partner’s lack of circumcision (RR = 1.9, 95% CI: 1.1–3.5, *P* = 0.03), Table 4.

**Table 4:** Factors associated with hr.-HPV infection in bivariate analyses, stratified by HIV serostatus

VARIABLES	HIV-1 seropositive women			HIV seronegative women		
	hr-HPV (*N* = 105) n (%)	RR (95% CI)	*P*-value	hr-HPV (*N* = 51) n (%)	RR (95% CI)	*P*-value
Age, years						
≤ 29	10 (90.9)	1		6 (30.0)	1	
30–59	89 (64.0)	0.6 (0.5–0.7)	<0.0001	38 (29.5)	1.1 (0.5–2.1)	0.94
> 60	6 (60.0)	0.5 (0.3–0.9)	0.02	7 (35.0)	1.2 (0.5–3.2)	0.64
Age at first sex, years categorical						
≤ 18	48 (57.8)	1		22 (29.3)	1	
> 18	57 (74.0)	1.3 (1.1–1.6)	0.03	29 (30.9)	1.1 (0.7–1.7)	0.83
Lifetime number of sexual partners						
1	9 (81.8)	1		8 (25.8)	1	
≥ 2	96 (64.4)	0.9 (0.6–1.2)	0.49	43 (31.2)	1.2 (0.6–2.3)	0.57
Circumcision status of main/current partner						
Circumcised	97 (64.7)	1		45 (28.5)	1	
Not circumcised	8 (80.0)	1.4 (1.2–1.6)	<0.0001	6 (54.6)	1.9 (1.1–3.5)	0.03
Smoking history						
Never smoked	101 (65.2)	1		50 (29.9)	1	
Smokers (Ex/current)	4 (80.0)	1.2 (0.8–1.9)	0.38	1 (50.0)	1.7 (0.9–3.8)	0.47
Hormonal contraceptive history						
Ever used	40 (63.5)	1		23 (32.4)	1	
Never used	65 (67.0)	1.1 (0.8–1.3)	0.66	28 (28.6)	0.9 (0.6–1.4)	0.63

### Prevalence of, and risk factors associated with abnormal cytology

**Fig. 1:**
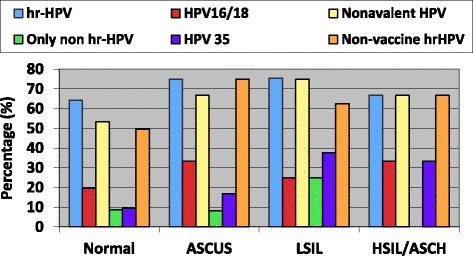
High-risk HPV genotypes prevalence by cytological category, for HIV-1 seropositive women (*N* = 163). Figure legend: ASCUS = Atypical Squamous Cells of Undetermined Significance, LSIL = Low Grade Squamous Intraepithelial Lesions, HSIL = High Grade Squamous Intraepithelial Lesions

The distribution of cytological results by HIV serostatus is shown in Table 1. The prevalence of abnormal cytology was higher among HIV-1 seropositive women (any SIL: 14.1% vs. 1.2%, *P* < 0.0001; low-grade SIL [LSIL]: 4.9% vs. 0.6%, *P* = 0.02; high-grade SIL [HSIL]: 1.8% vs. 0%, *P* = 0.07). HPV35 was the most frequent hr.-HPV type associated with LSIL and above (LSIL+) (Fig. 1).

Among HIV-1 seropositive women, having more than 4 pregnancies increased the risk of having LSIL+ abnormalities (aOR = 4.1, 95% CI: 1.1–17.0), while CD4+ count >350 cells/mm^3^ decreased the risk (aOR = 0.3, 95%CI: 0.07–0.9) (Table 5). HPV35 was the type most strongly associated with LSIL+, adjusting for age and HIV status (aOR = 4.7, 95% CI: 1.3–17.7, *P =* 0.02). There were too few HIV-seronegative women with SIL to conduct any meaningful risk factor analysis.

**Table 5:** Bivariate and multivariate analyses of low-grade squamous intraepithelial lesions and higher (LSIL+) among HIV-1 seropositive women in Cape Coast, Ghana

VARIABLES	LSIL+, n (%)	OR (95% CI)	*P*-value	^a^AOR (95% CI)	*P*-value
Age, years					
≤ 44	7 (7.9)	1		0.5 (0.1–1.8)	0.34
≥ 45	4 (5.4)	0.6 (0.2–2.4)	0.53		
Education					
≤ 6 years of formal education	7 (9.7)	1		0.5 (0.2–1.9)	0.30
> 6 years of formal education	4 (4.4)	0.4 (0.1–1.5)	0.18		
Employment					
Unemployed	2 (14.3)	1			
Employed	9 (6.0)	0.4 (0.1–2.0)	0.24		
Smoking history					
Never smoked	10 (6.3)	1			
Smokers (Ex/current)	1 (20.0)	3.7 (1.4–16.9)	0.23		
Number of pregnancies					
≤ 4	3 (3.3)	1		4.1 (1.1–17.0)	0.05
> 4	8 (11.1)	3.7 (0.9–14.7)	0.05		
Nadir CD4+ count, cells/mm^3^					
≤ 350	10 (8.9)	1		0.3 (0.07–0.9)	0.04
> 350	1 (2.3)	0.2 (0.03–2.0)	0.15		

^a^adjusted for age, level of education, total number of pregnancies and nadir CD4 count

## Discussion

This study reports for the first time the comparative prevalence and genotype distribution of HPV among HIV-1 seropositive and seronegative women in Ghana. Findings from research conducted mainly in developed countries and few studies across Africa have shown a higher prevalence of HPV infection (any HPV, hr.-HPV, multiple HPV) among HIV-1-seropositive compared with HIV-seronegative women [7, 35–37]. In Ghana as elsewhere, HIV-1 seropositive women were significantly more frequently infected with HPV, and twice more likely to have high-risk and multiple HPV genotypes. The absolute hr.-HPV prevalence among HIV-1 seropositive women (66.8%) is much higher than that reported by Ezechi et al. [36] in a study conducted among 220 HIV-1 seropositive and 295 HIV-seronegative women in Nigeria, which reported hr.-HPV prevalence of 24.6% and 15.9% in the respective groups. Another study in South Africa reported a twice higher prevalence of any hr.-HPV infection among 145 HIV-1 seropositive and 107 HIV-seronegative women (68% versus 31%) [38], which is comparable to this study. Some of the differences in prevalence may be attributable to the HPV detection assay used [39], with genotyping assays such as the Anyplex-II HPV 28 used in this study more likely to have higher sensitivity. In addition, the population in this study had relatively low nadir CD4+ count (<350 cells/mm^3^) and a worse WHO HIV/AIDS score III/IV (50%) hence more likely to have higher HPV prevalence.

### HPV genotype prevalence and associated risk factors

Since protection afforded by HPV vaccines is essentially type specific [40, 41], knowledge of the genotype distribution in a specific population has implications for vaccine choice and predicted impact. The most prevalent hr.-HPV type in both groups in this study was HPV35, which none of the available vaccines on the market includes. The quadrivalent HPV vaccine is used in most countries and covers for only HPV6, 11, 16 and 18 [42, 43]. The recent introduction of the nonavalent vaccine to cover for additional HPV31, 33, 45, 52 and 58 may lead to a change in vaccine choice among countries and countries yet to implement wide scale or routine vaccination programs including Ghana.

HPV16 and 18 are said to contribute to about 70% of cervical cancer cases [44] and this has influenced vaccine development. This study demonstrated the association between HPV35 and abnormal cytology in Ghana. A study in neighbouring Burkina Faso found HPV35 to be the second most prevalent among sex workers with high prevalence of HIV [37]. Another study conducted in South Africa reported HPV35 as the third most prevalent genotype identified among 154 HIV-seronegative women with ICC [45]. Pirek et al. [46] found HPV35 the fifth commonest among Cameroonian women with ICC and HPV45 was the second most prevalent type. Maranga et al. [47] in their study among Kenyan women also found that HPV45 contributed significantly to cervical cancer among HIV-seropositive women. Attoh et al.*,* [25] in their study among Ghanaian women with cervical cancer detected 8 h-HPV genotypes (16, 18, 35, 39, 45, 52, 56 and 66) with HPV18 being the most prevalent. Another study based in sub-Saharan Africa with inclusion of samples from Ghana found that HPV type distribution appeared to differ according to tumor type and HIV status and HPV16, 18, 45 and 35 were the most common HPV types in women with ICC [48]. In this study, hr.-HPV types included in the bi- or quadrivalent vaccines (HPV16/18) and nonavalent vaccine (HPV16/18/31/33/45/52 and 48) were found in higher proportions among HIV-1 seropositive compared with HIV-seronegative women.

Among study participants, there was evidence of an association between having hr.-HPV infection and younger age among HIV positive participants and lack of circumcision of the male partner among both HIV positive and negative groups. These findings are consistent with existing literature [49, 50]. Other studies in Ghana and other parts of the world have reported association between employment status, marital status and educational level with HPV [51, 52].

### Epidemiology of cytological abnormalities and associated risk factors

Compared with HIV-seronegative women, HIV-1 seropositive women in this study had a higher prevalence of SIL and higher grade cytological lesions. Indeed, HSIL/ASC-H were only identified among HIV-1 seropositive women. A study conducted among women in selected communities in the Ashanti region of Ghana found any SIL prevalence ranging between 3.5% and 12.6% [53], although this study did not report HIV status. Elsewhere in sub-Saharan Africa, Hood et al. reported that cervical lesions were significantly associated with the detection of plasma HIV RNA (with an adjusted relative risk of 1.16, 95% CI: 1.05–1.28) among women in Senegal [54]. Another study in Kenya among 267 HIV-seropositive women on ART found a much higher prevalence of abnormal cytology of 46%. The median duration of antiretroviral therapy was 13 months (IQR: 8–19) [55]. In another study conducted in South Africa among 109 HIV-seropositive women before initiation of ART, the prevalence of abnormal cytology was 66.3%. The median CD4 count among these women was very low at 125 cells/mm^3^ [56].

In the sub-group analysis for HIV-1 seropositive women, factors which showed strong evidence of an association with SIL included nadir CD4+ T-cell count. A higher CD4+ T-cell count reflects a stronger immune system, which may be associated with greater ability to clear HPV infection compared with women who acquire HPV whilst more seriously immunocompromised, which may lead to more frequent persistence and a greater chance to develop lesions. The association with nadir CD4+ T-cell count has been demonstrated in other studies [5, 6, 36]. This study had some limitations. It was facility-based, which allowed for more convenience in recruitment, but its results may not be generalizable. Histology confirmation of lesions would have also been preferable as it is more specific diagnosis than cytology, although this has been a usual limitation of several epidemiological studies in sub-Saharan Africa. An important limitation is the unavailability of current HIV viral load and CD4 count for the HIV positive participants. Nonetheless, this study brings useful information for decision makers in the country, demonstrating that the burden of HPV infection and hr.-HPV infection is high in Ghana. The study also showed that the most prevalent HPV genotype was HPV35, and that among HIV-seronegative women, HPV16 and 18 were not among the top five most prevalent genotypes. The established contribution from HPV 16 and 18 has appropriately informed vaccine choices, nevertheless, the genotype distribution pattern found in this and other studies imply the need to look carefully at vaccine choices for primary prevention of HPV and cervical cancer among women in Ghana.

## Conclusion

The study shows the need for a systematic national cervical cancer screening program in view of the high HPV prevalence even among the HIV seronegative women. This may include scheduled screening for women in Ghana using Pap smear, visual inspection (VIA) and the possibility of HPV screening. For HIV positive women, screening using HPV testing may be challenging since over 50% of them may be positive. Strong cervical cancer prevention and screening programs should be specifically deployed targeting HIV-seropositive women in Ghana. In addition, it demonstrates that prophylactic vaccination of young girls with the nonavalent HPV vaccine is likely the best means of cervical cancer prevention in Ghana.

## Additional file

Additional file 1: Questionnaire for collecting data from study participants. This is the questionnaire which was administered to all recruited women before sample collection towards this epidemiology study. (DOCX 51 kb)

## Abbreviations

ART: Anti-Retroviral Therapy
ASCUS: Atypical Squamous Cells of Undetermined Significance
CCTH: Cape Coast Teaching Hospital
CD4 +: Activated T-lymphocytes CD4
CI: Confidence Interval
CIN: Cervical Intraepithelial Neoplasia
HIV: Human Immunodeficiency Virus
HPV: Human Papilloma Virus
hr.-HPV: High-Risk Human Papilloma Virus
H-SIL: High Grade Squamous Intraepithelial Lesion
ICC: Invasive cervical carcinoma
KNUST: Kwame Nkrumah University of Science and Technology
LSIL: Low Grade Squamous Intraepithelial Lesion
PLHIV: People Living with HIV
SIL: Squamous Intraepithelial Lesion
SSA: Sub Saharan Africa
STI: Sexually Transmitted Infection
VIA: Visual Inspection with Acetic acid
VILI: Visual Inspection with Lugol’s iodine
WHO: World Health Organization
WLHA: Women Living with HIV/AIDS
