# Supplementary material for: Epidemiology of cervical human papillomavirus (HPV) infection and squamous intraepithelial lesions (SIL) among a cohort of HIV-infected and uninfected Ghanaian women
Source: BMC Cancer. 2017 Oct 16;17:688. doi: 10.1186/s12885-017-3682-x (PMC5644111; doi:10.1186/s12885-017-3682-x)
Supplement: Additional file 1: — Questionnaire for collecting data from study participants. This is the questionnaire which was administered to all recruited women before sample collection towards this epidemiology study. (DOCX 51 kb) [file 12885_2017_3682_MOESM1_ESM.docx]

**APPENDIX A: QUESTIONNAIRE FOR COLLECTING DATA FROM STUDY PARTICIPANTS**

| **DATE OF INTERVIEW:** | | | **STUDY CODE *(pre printed)*:** | **PARTICIPANT CODE:** | |
| --- | --- | --- | --- | --- | --- |
| **NAME OF INTERVIEWER:** | | | **SIGNATURE:** |  |  |
| **SOCIO-DEMOGRAPHICS** | | | | | |
| **No.** | **QUESTIONS** | | **CODE** | **RESPONSE(S)** | |
| **Q1** | **AGE** | | **(*write the actual number down)*** |  | |
| **Q2** | **OCCUPATION** | | 1. **Unemployed** 2. **Unskilled occupation** 3. **Skilled occupation** |  | |
| **Q3** | **RELIGION** | | 1. **Christian** 2. **Moslem** 3. **Traditional** 4. **Others** |  | |
| **Q4** | **MARITAL STATUS** | | 1. **Currently with a regular sexual partner** 2. **Currently without a regular sexual partner** |  | |
| **Q5** | **LEVEL OF EDUCATION** | | 1. **No formal education** 2. **Up to secondary school level** 3. **Tertiary level** |  | |
| **Q6** | **HOW MANY PREGNANCIES HAVE YOU HAD IN THE PAST?** | | **(*write the actual number down) If the answer is 0 then skip to Q10*** |  | |
| **Q7** | **HOW MANY OF THESE PREGNANCIES RESULTED IN STILLBIRTH?** | | **(*write the actual number down)*** |  | |
| **Q8** | **HOW MANY OF THESE PREGNANCIES RESULTED IN MISCARRIAGE/ABORTION?** | | **(*write the actual number down)*** |  | |
| **Q9** | **HOW MANY LIVE CHILDREN DO YOU HAVE?** | | **(*write the actual number down)*** |  | |
| **RISK FACTORS** | | | | | |
| **Q10** | **HIV STATUS (confirmed status)** | 1. **Positive** 2. **Negative *(jump to question Q12)*** | | |  |
| **Q11** | **HOW LONG HAS IT BEEN SINCE YOU WERE DIAGNOSED?** | **(*write the duration as much as possible in months)*** | | |  |
| **Q12** | **HOW MANY SEXUAL PARTNERS HAVE YOU HAD IN YOUR WHOLE LIFE?** | **(*write the actual number down)*** | | |  |
| **Q13** | **HOW OLD WERE YOU WHEN YOU HAD YOUR FIRST SEXUAL INTERCOURSE?** | ***(Write the actual age)*** | | |  |
| **Q14** | **WHAT IS THE HIV STATUS OF YOUR MAIN/CURRENT SEXUAL PARTNER?** | 1. **Positive** 2. **Negative** 3. **Don’t know** | | |  |
| **Q15** | **IS YOUR MAIN/CURRENT SEXUAL PARTNER(S) CIRCUMCISED?** | 1. **Yes** 2. **No** 3. **Don’t know** | | |  |
| **Q16** | **HAVE YOU EVER USED ANY HORMONAL CONTRACEPTIVE BEFORE?** | 1. **Yes** 2. **No** | | |  |
| **Q17** | **HAVE YOU EVER SMOKED CIGARETTES?** | 1. **Yes** 2. **No** | | |  |
| **DATA FROM CLIENT BOOKLET FOR HIV POSITIVE CLIENTS** | | | | | |
| **Q18** | **WHO CLINICAL STAGE** | | **(*Write in 1,2,3,4)*** |  | |
| **Q19** | **ART STATUS?** | | 1. **Not on ART** 2. **≤ 2 years on ART** 3. **>2 years on ART** |  | |
| **Q20** | **CD4 COUNT*( nadir)* Cells/mm^3^** | | ***Write actual value*** |  | |
| ***THANK YOU*** | | | | | |
